# Aflatoxin profiles of *Aspergillus flavus* isolates in Sudanese fungal rhinosinusitis

**DOI:** 10.1093/mmy/myae034

**Published:** 2024-04-05

**Authors:** Shaoqin Zhou, Mawahib A I Ismail, Vishukumar Aimanianda, G Sybren de Hoog, Yingqian Kang, Sarah A Ahmed

**Affiliations:** School of Public Health, Key Laboratory of Environmental Pollution Monitoring and Disease Control, Ministry of Education of Guizhou, Key Laboratory of Microbiology and Parasitology of Education Department of Guizhou, School of Basic Medical Science, Guizhou Medical University, 561113, Guiyang, China; Radboudumc-CWZ Centre of Expertise for Mycology, 6525 GA, Nijmegen, The Netherlands; Mycology Reference Laboratory, University of Khartoum, 11115, Khartoum, Sudan; Immunobiology of Aspergillus, Institut Pasteur, Universite ´ Paris Cite ´ 75015, Paris, France; Radboudumc-CWZ Centre of Expertise for Mycology, 6525 GA, Nijmegen, The Netherlands; Foundation Atlas of Clinical Fungi, 1214 GP, Hilversum, The Netherlands; School of Public Health, Key Laboratory of Environmental Pollution Monitoring and Disease Control, Ministry of Education of Guizhou, Key Laboratory of Microbiology and Parasitology of Education Department of Guizhou, School of Basic Medical Science, Guizhou Medical University, 561113, Guiyang, China; Radboudumc-CWZ Centre of Expertise for Mycology, 6525 GA, Nijmegen, The Netherlands; Foundation Atlas of Clinical Fungi, 1214 GP, Hilversum, The Netherlands

**Keywords:** *Aspergillus flavus*, fungal rhinosinusitis, aflatoxin profiles, thin-layer and high-performance chromatography, gene expression

## Abstract

*Aspergillus flavus* is a commonly encountered pathogen responsible for fungal rhinosinusitis (FRS) in arid regions. The species is known to produce aflatoxins, posing a significant risk to human health. This study aimed to investigate the aflatoxin profiles of *A. flavus* isolates causing FRS in Sudan. A total of 93 clinical and 34 environmental *A. flavus i*solates were studied. Aflatoxin profiles were evaluated by phenotypic (thin-layer and high-performance chromatography) and genotypic methods at various temperatures and substrates. Gene expression of *aflD* and *aflR* was also analyzed. A total of 42/93 (45%) isolates were positive for aflatoxin B1 and AFB2 by HPLC. When the incubation temperature changed from 28°C to 36°C, the number of positive isolates decreased to 41% (38/93). Genetic analysis revealed that 85% (79/93) of clinical isolates possessed all seven aflatoxin biosynthesis-associated genes, while 27% (14/51) of non-producing isolates lacked specific genes (*aflD/aflR*/*aflS*). Mutations were observed in *aflS* and *aflR* genes across both aflatoxin-producers and non-producers. Gene expression of *aflD* and *aflR* showed the highest expression between the 4th and 6th days of incubation on the Sabouraud medium and on the 9th day of incubation on the RPMI (Roswell Park Memorial Institute) medium. *Aspergillus flavus* clinical isolates demonstrated aflatoxigenic capabilities, influenced by incubation temperature and substrate. Dynamic *aflD* and *aflR* gene expression patterns over time enriched our understanding of aflatoxin production regulation. The overall findings underscored the health risks of Sudanese patients infected by this species, emphasizing the importance of monitoring aflatoxin exposure.

## Introduction

*Aspergillus flavus*, a common airborne species, is frequently encountered in agricultural settings, where it is known for contaminating crops, particularly grains and peanuts. However, its significance extends beyond agriculture, as it plays a pivotal role in causing invasive aspergillosis (IA), notably fungal rhinosinusitis (FRS).^1,2^ This fungus is responsible for 86% of FRS cases in Sudan,^3^ highlighting its importance in arid regions compared to *A. fumigatus*, the global primary cause of IA and a top concern on the WHO's fungal priority pathogens list. FRS places a significant burden in arid regions,^4^ with around 1.5 million cases annually reported in India and an estimated incidence of 200 cases per 100 000 in Sudan.^5,6^ An elevated incidence rate has also been observed beyond arid regions, evidenced by 392 cases per 100 000 individuals documented in Turkey.^7^ Notably, epidemiologic data on the disease in other countries of the world is scarce. FRS encompasses two distinct forms: invasive and non-invasive. The latter is milder and often chronic, primarily confined to the sinuses, while the invasive form is severe, but can also manifest as a chronic condition that can last for years.^8^

*Aspergillus flavus* is notorious for producing toxic metabolites and primarily mycotoxins that pose a severe threat to human health. Aflatoxin B1 (AFB1), a prominent mycotoxin produced by *A. flavus*, has been classified as a Group 1 carcinogen by the International Agency for Research on Cancer (IARC).^9^ Prolonged exposure to AFB1 can compromise cell-mediated immunity, increasing susceptibility to various diseases.^10^ A study conducted in Japan demonstrated that aflatoxin could suppress leukocyte function and immune mechanisms, further emphasizing its detrimental effects.^11^ Although the detection of aflatoxin in humans *in vivo* has not been extensively explored, two studies in Japan have reported the presence of aflatoxin in autopsied materials from patients infected with *A. flavus*,^11,12^ providing compelling evidence that the species can indeed produce aflatoxin *in vivo*. Given the capacity of *A. flavus* to produce aflatoxins, which can be chronically exposed to humans, there might be a potential influence on pathogenicity, and to possible predisposition of chronically infected individuals to nasopharyngeal carcinoma. Consequently, the investigation of the regularity of aflatoxin production and a deeper exploration of the mechanism governing this production in clinical settings remains imperative.

Aflatoxins are products of a complex biosynthesis pathway involving a minimum of 27 enzymatic reactions, known as the polyketide pathway.^13^ The genes responsible for encoding these enzymes are clustered, encompassing a total of 30 genes, and their expression is coordinated by two cluster-specific regulators: *aflR* and *aflS*.^14,15^ While *aflR* governs both aflatoxin and sterigmatocystin production, *aflS* (also known as *aflJ*) is exclusively associated with aflatoxin regulation. A key structural gene in the biosynthetic pathway, *aflD* (*nor-1*), encodes an enzyme responsible for catalyzing the conversion of the initial aflatoxin biosynthesis intermediate, norsolorinic acid, to averantin.^16^ Additional other key genes include *aflM* (*ver-1*), *aflO* (*omtB*), and *aflP* (*omtA*), among others.^17^ A series of studies have indicated that the expression of *aflR, aflD*, and *aflS* can serve as reliable markers and correlate with aflatoxin production.^18–21^*AflR*, in particular, a positive regulator of the aflatoxin gene cluster, has been demonstrated to induce a significant upregulation, resulting in a 50-fold increase in aflatoxin production in *A. flavus*.^22^ Furthermore, studies have shown that overexpression of *aflR* could influence the transcriptional and aflatoxigenic patterns in *A. flavus*.^23^ Notably, expression of those genes is largely affected by ecological factors and nutritional conditions, with studies indicating that climate changes, particularly exposure to elevated CO_2_ concentrations, can significantly upregulate *aflD* and *aflR* gene expression resulting in higher production of toxin.^24,25^ While these studies addressed aflatoxin production in agricultural contexts, comprehensive investigations focusing on strains from clinical sources remain limited. Analysis of the aflatoxin characterization of clinical *A. flavus* may contribute to the understanding of pathogenicity and disease progression.

Several methods are currently employed for aflatoxin detection, including thin-layer chromatography (TLC), enzyme-linked immunosorbent assay (ELISA), high-performance liquid chromatography with fluorescence detector (HPLC-FLD) or with tandem mass spectrometry (HPLC-MS/MS). TLC and ELISA are widely utilized to detect aflatoxins in various foods, offering sensitivity around 1–20 µg/kg and higher sensitivity but lower stability, respectively.^26,27^ Currently, HPLC with fluorescence detection (HPLC-FLD) is the most popular chromatographic technique for the separation and determination of aflatoxin, with a limit of detection (LOD) as low as 0.1 ng/kg.^28–30^ A comprehensive analysis of aflatoxins can be achieved by combining quantitative and qualitative methods.

Our investigation aimed to explore aflatoxin production in clinical and environmental isolates. An assessment was conducted to investigate the impact of different environmental variables, including temperature and growth media on aflatoxin production, by employing both qualitative and quantitative methods. Furthermore, aflatoxigenic genes were detected, thereby providing insights into the genetic mechanisms behind aflatoxin biosynthesis and gene expression profiles of *aflR* and *aflD*, with particular emphasis on settings that simulate the host environment.

## Materials and methods

### Strains source, isolation, and identification of environmental strains

Ninety-three clinical *Aspergillus flavus* isolates (88 clinical isolates have been published previously^3^ and five are shown in Supplementary Table 1) were acquired from patients diagnosed with FRS in Sudan maintained in 20% glycerol solution and stored at −80°C. To enable a comparison between clinical and environmental sources, environmental isolates were originally designed to be collected from local surroundings. Unfortunately, access to the isolates from Sudan was not possible due to safety concerns. Therefore, *A. flavus* from other geographic regions were included as follows: 16 were extracted from crops, such as peanuts and corn, in China, 13 were obtained from the CBS reference collection (origin: England, Japan, South Africa, Uganda, and Brazil), and five were achieved from the Mycology Reference Laboratory at Radboudumc (The Netherlands). As negative controls for aflatoxin detection, three isolates (*A. terreus, Penicillium citrinum*, and *Trichoderma asperellum*) were included. Identification of the isolates involved a combination of morphological^31^ and molecular analysis (GenBank accession number shown in Supplementary Table 1),^32^ comprising sequencing of the ITS (Internal transcribed spacer) rDNA region, a segment of the *BenA* gene encoding β-tubulin,^33^ and partial *CaM* gene encoding calmodulin.^34^

### Morphology and growth

Spore suspensions were prepared at a concentration of 1 × 10^6^ spores/ml using a hemocytometer and observed with a light microscope (Zeiss Axio Scope, Carl Zeiss, Göttingen, Germany). A volume of 20 μl of the spore suspension was inoculated centrally on 9-cm Petri dishes with various media, including Sabouraud glucose agar (SDA, Thermo Fisher Scientific, MA, USA), Water agar (WA), RPMI agar, WA + 4% glucose, and Blood agar (BA: containing 5% sheep blood). Three replicates per sample were incubated under two distinct temperatures (28 ± 1°C and 36 ± 1°C) for 6 days. Micro-morphology was examined using a light microscope (Zeiss Axio Scope, Carl Zeiss, Göttingen, Germany). The images were annotated in Adobe Photoshop 2022. Diameters of the colonies were recorded after the termination of the incubation to compare the growth on different media. These cultures were applied for aflatoxin detection following the observation of morphology and growth.

### Aflatoxin detection by TLC and HPLC

#### Aflatoxin extraction

For aflatoxin extraction, agar plugs (5 plugs per plate, approx. 0.5 g) were excised using a five-point sampling method.^21^ Those plugs were then mixed with 600 µl methanol (high grade) and subjected to 30 min of shaking using a TissueLyser (Xinyi-48, Ningbo, China). After centrifugation at 6000 rpm for 10 min, the supernatant was carefully transferred to a new tube.^35^ The extracts were directly filtered through a Nylon 13 mm, 0.22 µm filter (BKMAM-Lab Ltd, Changde, China) into amber HPLC vials for further analysis.

#### Quantitative analysis by TLC

Silica gel-coated plates (100 mm × 200 mm, GF254) were prepared with a marked origin line approximately 3 cm from the bottom. Standard samples of AFB1/AFB2/AFG1 and AFG2 (1 μg/ml) were spotted on the origin line and placed in development tanks containing a development solution [chloroform: acetone 92:8 (v/v)] for a duration of 30 min. Plates were then examined under UV light (WFH-203B, Shanghai, China) to detect aflatoxins based on characteristic fluorescence.^35,36^

#### Qualitative analysis by HPLC

Standard stock solutions (10 μg/ml) were prepared. A mixture of standard working solution (AFB1 and AFG1: 100 ng/ml, AFB2 and AFG2: 30 ng/ml), as well as a standard series working solution were prepared in accordance with the standards outlined in the aflatoxin detection protocol (GB 5009.22-2016, https://food.chemlinked.com/database/view/555). Sample extracts were subsequently subjected to HPLC using an Ultimate 3000 system controller, 470 fluorescence detector (Thermo Scientific, MA, USA) (lexc, 360 nm; lem, 450 nm) and a C18 column (Agilent, 250 mm × 4.6 mm, 5 µm) all controlled by Ultimate 3000 software. A 50 µl of each sample was injected and column temperature was maintained at 35°C. The analysis was performed at a flow rate of 1 ml/min of the mobile phase (40% methanol: 60% water) and the run time was set at 30 min. Post-column photochemistry derivatization (Ruipin Biology Ltd. Shanghai, China) was applied to the samples. Standard series working solutions were injected into HPLC to establish a standard curve (Supplementary Table 2). The detection limit (LOD) and quantification limit (LOQ) were established using the standard deviation (SD) of the response (Sb) and the calibration curve's slope (m), as outlined by LOD = 3 Sb/m, LOQ = 10 Sb/m (Supplementary Table 2).

### Detection of aflatoxin biosynthesis-associated genes

Seven aflatoxin biosynthesis-associated genes (*aflD, aflM, aflO, aflP, aflQ aflR*, and *aflS*) were detected. DNA extraction, PCR (Polymerase Chain Reaction) amplification and gel electrophoresis of all isolates were performed following standard procedures.^37^ The seven pairs of primers used were in accordance with the specification outlined by Gallo et al.^38^ PCR products of 10 randomly selected aflatoxin-producing isolates and 10 non-aflatoxin-producing isolates were chosen for Sanger Sequencer-based sequencing. Subsequently, the acquired sequences were assembled using SnapGene and aligned with the reference strains through Benchling (https://www.benchling.com/) (GenBank accession numbers shown in Supplementary Table 3). The graph of gene presence *vs*. absence was presented by chiplot (https://www.chiplot.online/).

### Relative gene expression of *aflD* and *aflR*

#### Strain incubation

Ten high-level aflatoxin-producing *A. flavus* isolates including five from the environment and five from clinical origin were selected for gene expression study. Additionally, two aflatoxin-non-producing isolates that possessed all genes associated with aflatoxin biosynthesis were also included for comparison. The spore suspension was prepared as described previously. Each spore suspension (1 ml) was cultured in 12-well microplates using Sabouraud dextrose broth (SDB, Thermo Fisher Scientific, MA, USA) and RPMI 1640. All microplates were cultured under normal CO_2_ (0.04%) and 5% CO_2_ incubation conditions at 36 ± 1°C for durations of 2, 4, 6, 9, and 12 days, respectively. Experiments were conducted in triplicate. RPMI 1640 was selected due to its common use in clinical research, and the 5% CO_2_ concentration aimed to mimic human tissue.

#### RNA extraction and cDNA

Biomass harvested from the cultures was stored at −80°C until RNA extraction. RNA was extracted using Trizol reagents (Thermo Fisher Scientific, MA, USA) and purified following established protocols.^39^ RNA concentration and quality were determined using a Nanodrop spectrophotometer (Thermo Fisher Scientific, Wilmington, DE, USA). Reverse-transcription PCR (RT-PCR) was carried out using a two-step reverse-transcription real-time quantitative PCR (qPCR) kit. Initially, cDNA was synthesized from 1000 ng of total RNA, following the PrimeScript RT Reagent kit protocol (New England BioLabs Inc.). The resulting cDNA served as template amplification using LightCycler® 480 SYBR Green I Master kit (Thermo Fisher Scientific, Wilmington, DE, USA).

#### qPCR analysis

The qPCR reactions were carried out using the Roche LightCycler® 480 II instrument (Roche Exiqon, Mannheim, Germany). The assays were prepared in triplicate in optical 96-well reaction plates and sealed with optical adhesive covers. The SYBR Green methodology was performed, with each reaction containing 10 µl of SYBR mix 2×, 0.5 µl of each 10 µM primer, and 5 µl of each cDNA template, resulting in a final volume of 20 µl. A negative control without cDNA was included in every run. The thermal cycling protocol consisted of an initial pre-incubation at 95°C for 5 min, followed by 45 cycles of 95°C for 10 s and 55°C for 20 s. Melting curve analysis was performed by heating to 95°C for 5 s, 65°C for 1 min and 97°C with continuous fluorescence measurement to quantify the PCR product. The threshold cycle (Ct) value was determined using LightCycler® 480 Software. Primer pairs F/R-*aflD, aflR* and β-tubulin were used to amplify the structural gene (*aflD*), the regulatory gene (*aflR*), and the housekeeping gene (*BenA*), respectively.^25^

Relative quantification of the expression of *aflD* and *aflR* genes was carried out using *BenA* gene. The expression ratio was calculated following the previous method.^40^ Log2 values of the relative expression of the *aflD* and *aflR* genes were graphically represented.

### Data analysis

The data analyses were conducted using analysis of variance (Anova) with the IBM SPSS 27 statistical analysis software. The results were reported as the mean ± SD. Statistically significant differences were observed by employing Duncan's novel multiple-range test at the significance levels of *P* = .05.

## Results

### Morphology and growth

**Figure 1.**
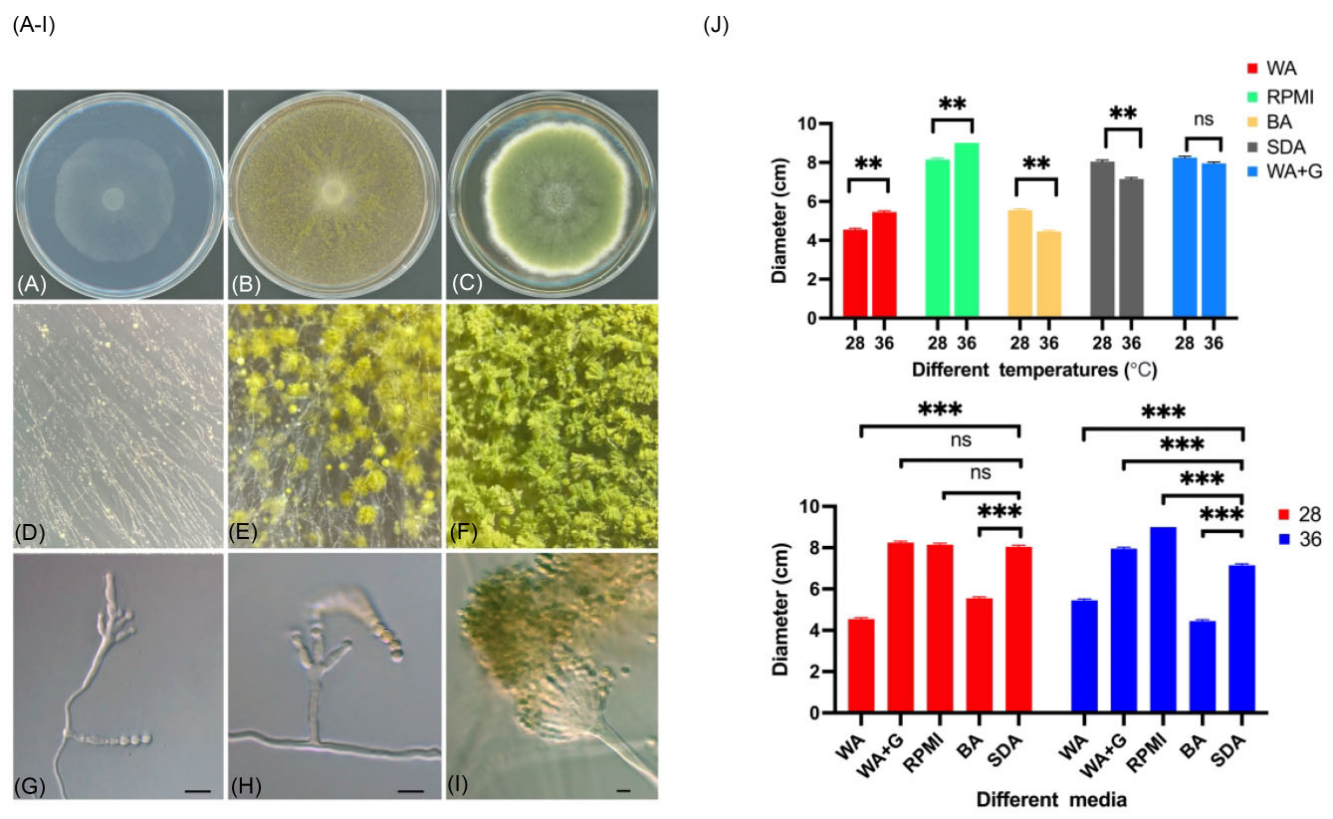
Morphology and growth of isolate M.064-44 cultured in different media at 28°C and 36°C for 6 days of incubation, respectively. (A–I) Macro- and micromorphology of isolates incubated at 36°C, first row: Colony, second row: Micromorphology, third row: Micromorphology of isolates incubated on WA, RPMI, and SDA from left to right; (J) The growth diameter of isolates. WA Water agar; WA + G Water agar with 4% glucose; BA Blood agar; SDA Sabouraud dextrose agar.

Isolates cultured on SDA showed yellow-green spores and typical conidia structure, with conidial emerging from the phialides located on the conidiophore vesicles (Fig. 1). On WA and RPMI agar, the isolates exhibited limited growth and degenerated sporulation, with observation of sparse conidia and an absence of characteristic *Aspergillus* head. Blood agar resulted in slow growth and a lack of sporulation, despite a notable increase in hyphal biomass thickness.

### Aflatoxin detection by TLC

**Figure 2.**
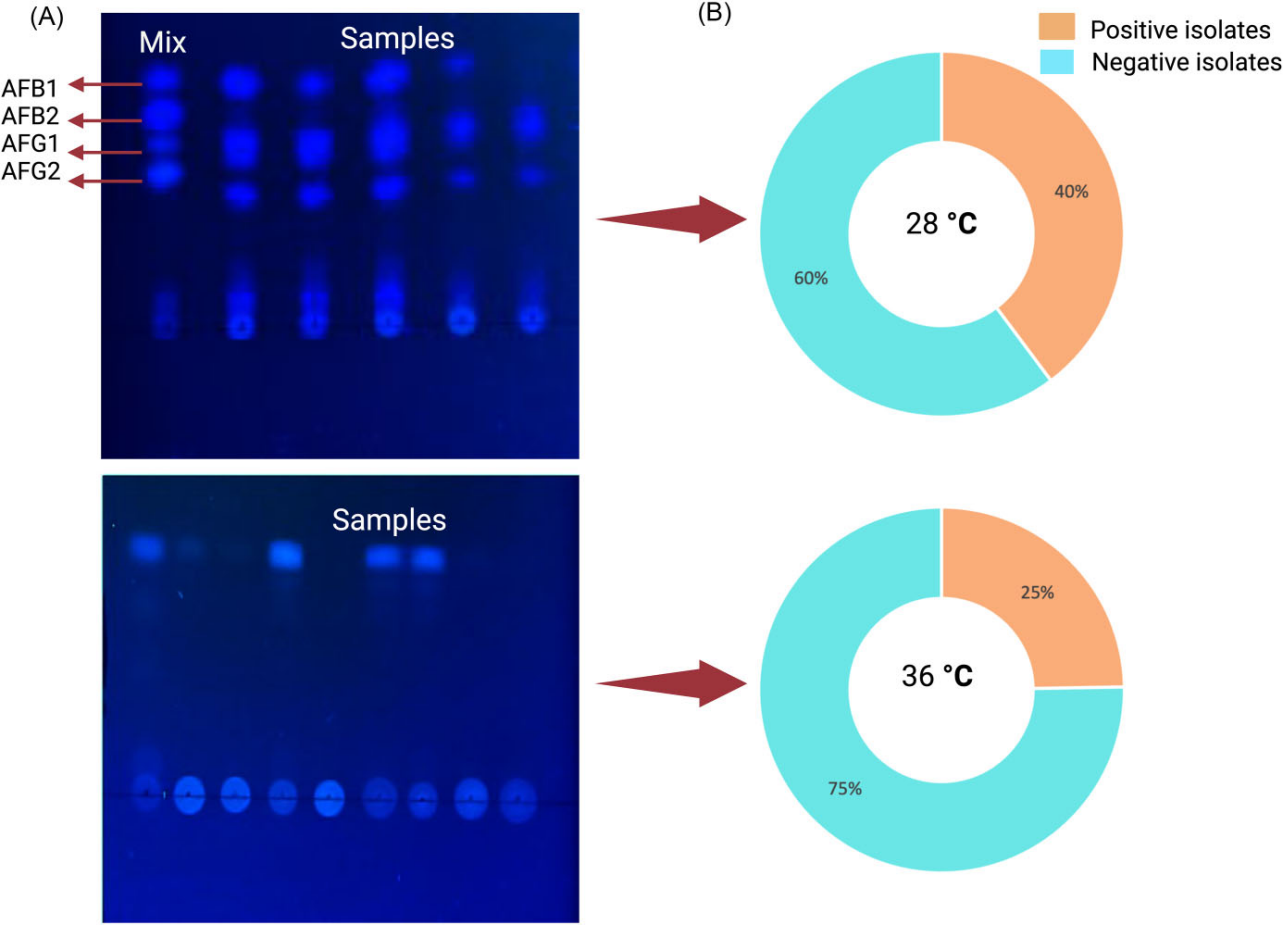
Aflatoxins of standard samples and clinical isolates detected by TLC. (A) Aflatoxins produced by clinical isolates incubated at 28°C. Mix – a mixture of standard aflatoxins, AFB1 – aflatoxin B1, AFB2 – aflatoxin B2, AFG1 – aflatoxin G1, and AFG2 – aflatoxin G2; (B) Aflatoxins produced by clinical isolates incubated at 36°C.

Of the 93 clinical isolates tested, 40% (37/93) demonstrated positive aflatoxin production when incubated on an SDA medium at 28°C. Incubation at 36°C resulted in a reduction in the proportion of aflatoxin-producing isolates, with only 25% (23/93) exhibiting positivity (Fig. 2, Supplementary Table 4). For the environmental isolates, 47% (16/34) displayed aflatoxin production at 28°C. The TLC proved to be somewhat constrained in its capacity to differentiate between various aflatoxin types, as certain isolates exhibited positive bands for all types, albeit with varying sizes. The aflatoxin-producing capacity of *Aspergillus flavus* isolates by TLC following 6 days of incubation revealed a lack of production among isolates when cultured on WA, WA + G, BA, and RPMI media.

### Aflatoxin detection by HPLC

**Figure 3.**
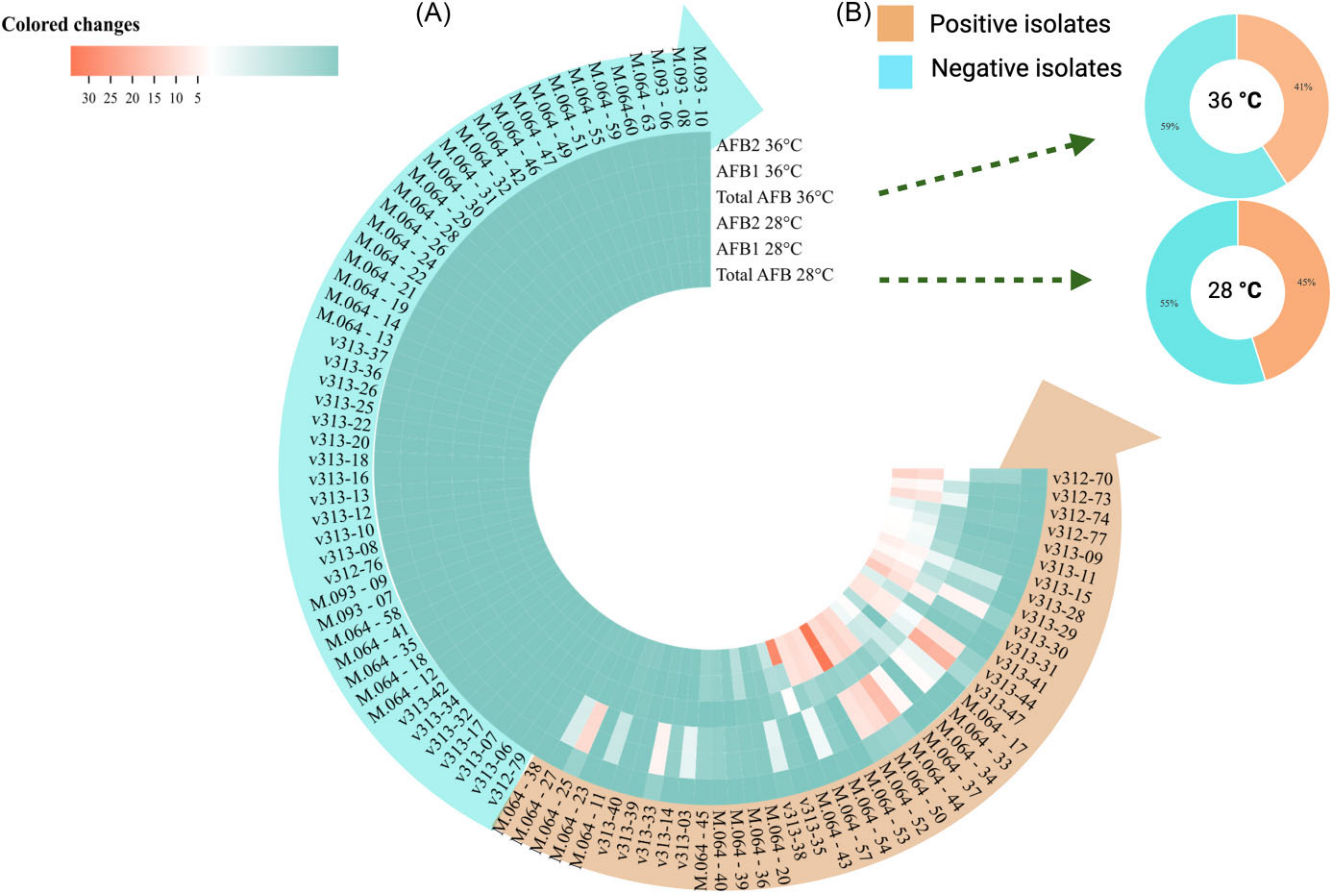
The proportion and amount of aflatoxins produced by *Aspergillus flavus* clinical isolates incubated at 28 and 36°C by HPLC. (A) The proportion of aflatoxins produced by clinical isolates and (B) the amount of aflatoxins.

Following the preliminary detection via TLC, all samples were subjected to HPLC analysis for a more comprehensive and quantitative evaluation of aflatoxin presence. The HPLC results remained consistent with those obtained through TLC and showed higher sensitivity. It was noteworthy that 45% (42/93) of clinical isolates and 50% (17/34) of environmental isolates were positive with HPLC, representing a significant increase of 5% (*P *< .001) and 3%, respectively, compared to TLC findings (Fig. 3B). Further analysis of the positive results unveiled that these isolates exclusively produced B-type aflatoxins, with no evidence of G-type. Among these isolates, the majority were capable of producing AFB1 and AFB2, although 12% (5/42) of clinical isolates exclusively synthesized AFB2, while 10% (4/42) exclusively produced AFB1 (Table 2), and 79% (33/42) of clinical isolates could generate both AFB1 and AFB2, with AFB1 levels significantly surpassing AFB2 at 28°C.

**Table 1.** Amount of aflatoxins detected by HPLC(mg/kg).

	AFB(mg/kg)					
	28°C			36°C		
Clinical isolates Radboudumc ID	Total AFB	B1	B2	Total AFB	B1	B2
v312-70	12.53 ± 0.03	10.54 ± 0.04	1.99 ± 0.05	0.51 ± 0.01	0.5 ± 0.01	0.01 ± 0
v312-73	4.2 ± 0.02	3.11 ± 0.01	1.09 ± 0.03	0.1 ± 0	0.1 ± 0	0
v312-74	9.61 ± 0.03	7.91 ± 0.03	1.7 ± 0.04	0.04 ± 0	0.04 ± 0	0
v312-77	1.69 ± 0	1.26 ± 0.01	0.43 ± 0.01	0.12 ± 0	0.11 ± 0	0.01 ± 0
v313-09	2.05 ± 0.02	1.57 ± 0.01	0.48 ± 0.01	0.01 ± 0	0.01 ± 0	0
v313-11	2.36 ± 0.01	1.85 ± 0	0.51 ± 0.01	0.11 ± 0	0.11 ± 0	0
v313-15	2.53 ± 0.01	1.79 ± 0	0.74 ± 0.02	0.17 ± 0	0.16 ± 0	0.01 ± 0
v313-28	5.81 ± 0.04	4.1 ± 0.01	1.71 ± 0.03	1.2 ± 0.02	1.2 ± 0.02	0
v313-29	3.56 ± 0.02	2.65 ± 0	0.9 ± 0.02	0.56 ± 0	0.54 ± 0	0.02 ± 0
v313-30	7.99 ± 0.02	6.82 ± 0.01	1.17 ± 0.02	4.71 ± 0.06	4.37 ± 0.06	0.34 ± 0
v313-31	14.32 ± 0.11	9.06 ± 0.09	5.27 ± 0.13	0.28 ± 0.01	0.28 ± 0.01	0.01 ± 0
v313-41	1.45 ± 0	1.1 ± 0.01	0.35 ± 0.01	0.06 ± 0	0.06 ± 0	0
v313-44	8.24 ± 0.04	6.34 ± 0	1.89 ± 0.04	8.36 ± 0.02	8 ± 0.02	0.36 ± 0
v313-47	8.3 ± 0.04	6.68 ± 0	1.62 ± 0.04	20.75 ± 0.13	20.12 ± 0.12	0.64 ± 0
M.064-17	0.85 ± 0.02	0	0.85 ± 0.02	1.66 ± 0.06	1.66 ± 0.06	0
M.064-33	2.54 ± 0.03	1.27 ± 0	1.27 ± 0.03	2.72 ± 0.02	2.72 ± 0.02	0
M.064-34	1.76 ± 0.01	1.19 ± 0	0.58 ± 0.01	0	0	0
M.064-37	10.26 ± 0.01	9.99 ± 0	0.26 ± 0	3.69 ± 0.02	3.69 ± 0.02	0
M.064-44	12.77 ± 0	12.42 ± 0.01	0.35 ± 0.01	17.9 ± 0.06	17.42 ± 0.06	0.47 ± 0
M.064-50	10.12 ± 0.02	9.66 ± 0.01	0.46 ± 0.01	10.63 ± 0.02	10.23 ± 0.02	0.4 ± 0.01
M.064-52	34.17 ± 0	33.83 ± 0.01	0.35 ± 0.01	8.54 ± 0.02	8.22 ± 0.03	0.32 ± 0.01
M.064-53	9.24 ± 0	8.88 ± 0.01	0.36 ± 0.01	0	0	0
M.064-54	10.69 ± 0.01	10.11 ± 0	0.58 ± 0.01	0.22 ± 0	0.21 ± 0	0.01 ± 0
M.064-57	8.79 ± 0.03	8.46 ± 0.04	0.33 ± 0.01	1.78 ± 0.03	1.78 ± 0.03	0
M.064-43	3 ± 0.06	0.71 ± 0	2.29 ± 0.06	0	0	0
v313-35	1.09 ± 0	0.76 ± 0.01	0.32 ± 0.01	0.15 ± 0	0.15 ± 0	0.001 ± 0
v313-38	0.27 ± 0	0.16 ± 0	0.11 ± 0	1.58 ± 0	1.51 ± 0	0.07 ± 0
M.064-20	0.17 ± 0	0.13 ± 0	0.04 ± 0	0.09 ± 0	0.09 ± 0	0
M.064-36	0.87 ± 0	0.82 ± 0	0.04 ± 0	0.11 ± 0	0.11 ± 0	0
M.064-39	0.18 ± 0	0.18 ± 0	0	0.17 ± 0	0.17 ± 0	0
M.064-40	0.32 ± 0	0.32 ± 0	0	0.32 ± 0	0.32 ± 0	0
M.064-45	0.39 ± 0	0.39 ± 0	0	0.38 ± 0.01	0.38 ± 0.01	0
v313-03	0.04 ± 0	0.02 ± 0	0.02 ± 0	1.62 ± 0.03	1.6 ± 0.03	0.02 ± 0
v313-14	0.03 ± 0	0	0.03 ± 0	0.31 ± 0	0.3 ± 0	0.01 ± 0
v313-33	0.06 ± 0	0.04 ± 0	0.02 ± 0	4.94 ± 0.04	4.59 ± 0.04	0.35 ± 0
v313-39	0.01 ± 0.02	0 ± 0	0.01 ± 0.02	0.03 ± 0	0.03 ± 0	0
v313-40	0.12 ± 0.06	0.03 ± 0	0.08 ± 0.06	0.05 ± 0	0.03 ± 0	0.01 ± 0
M.064-11	0.12 ± 0	0.1 ± 0	0.02 ± 0	1.14 ± 0.07	1.14 ± 0.07	0
M.064-23	0.01 ± 0	0	0.01 ± 0	0	0	0
M.064-25	0.02 ± 0	0	0.02 ± 0	11.06 ± 0.04	10.67 ± 0.03	0.39 ± 0.01
M.064-27	0.04 ± 0	0	0.04 ± 0	1.35 ± 0.02	1.35 ± 0.02	0
M.064-38	0.03 ± 0	0.03 ± 0	0	0.03 ± 0	0.03 ± 0	0
Environmental isolates						
M.093-12	0.53 ± 0.01	0.51 ± 0.01	0.02 ± 0	–	–	–
M.093-13	19.59 ± 0.22	19.37 ± 0.22	0.23 ± 0	–	–	
M.093-14	1.4 ± 0.01	1.38 ± 0	0.02 ± 0	–	–	–
M.093-15	2.04 ± 0.05	2.02 ± 0.05	0.02 ± 0	–	–	–
M.093-16	0.6 ± 0.71	0.6 ± 0.71	0.01 ± 0	–	–	–
M.093-17	2.98 ± 0.01	2.98 ± 0.01	0	–	–	–
M.093-18	1.87 ± 0.04	1.87 ± 0.04	0	–	–	–
M.093-19	2.36 ± 0	2.23 ± 0	0.13 ± 0	–	–	–
M.093-20	7.62 ± 0.01	7.28 ± 0.01	0.35 ± 0	–	–	–
M.093-27	4.45 ± 0	4.33 ± 0.01	0.12 ± 0	–	–	–
M.093-28	0.74 ± 0	0.72 ± 0	0.02 ± 0	–	–	–
M.093-29	0.65 ± 0	0.63 ± 0	0.02 ± 0	–	–	–
CBS119.62	0.05 ± 0	0.02 ± 0	0.02 ± 0	–	–	–
CBS816.96	0.04 ± 0	0.04 ± 0	0.003 ± 0	–	–	–
CBS117.62	0.37 ± 0	0.36 ± 0	0.003 ± 0	–	–	–
CBS118.62	0.45 ± 0	0.45 ± 0	0.003 ± 0	–	–	–
CBS501.65	0.002 ± 0	0.002 ± 0	0	–	–	–

HPLC high performance liquid chromatography; AFB aflatoxin B;-no detection.

**Table 2.** Mutations of aflatoxin biosynthesis associated genes (*aflD/aflR/aflR*) single nucleotide polymorphisms (SNP).

	Mutation (non-synonymous mutation/synonymous mutation)			
Strains number	a*flR*	*aflD*	*aflS*	Aflatoxin profiles
**NRRL 3375 Reference strains(mRNA)**	–	–	–	Positive
v312-70	K43E T106T V194A T270T S379S G399R (3/3)	–	A131A M135L L150L S159S S161S T165T G186G E193A S213S P253P G271G A315A (2/10)	Positive
v312-73	V194A (1/0)	–	M135L D147D L150L S159S E193A A218V M227M Q254Q R259R G271G T306I (4/7)	Positive
v312-74	–	–	E193A A315A (1/1)	Positive
v313-47	–	–	E193A L292L (1/1)	Positive
M.064-44	–	–	M135L D147D E193A Q254Q (2/2)	Positive
v312-76	V194A (1/0)	–	E193S (1/0)	Negative
v313-10	–	–	S133S M135L A192A E193A A315A (2/3)	Negative
v313-12	V194A (1/0)	–	S133S M135L A192A E193A A315A (2/3)	Negative
M.064-55	T106T P136P S184S V194A C265Y G327S M359V S379S (4/4)	D19D A68P T252T (1/2)	M135L S189S E193A A268T G271G A284A P291P A315A (3/5)	Negative
M.064-59	V194A (1/0)	–	E193S (1/0)	Negative

‘-’, Stands for ‘no mutation’.

The range of the total aflatoxin production for clinical isolates spanned from 0.01 ± 0 to 34.17±0 mg/kg at 28°C and 0.01 ± 0 to 20.75 ± 0.13 mg/kg at 36°C. In contrast, environmental isolates displayed a range of 0.002 ± 0 to 19.59 ± 0.22 mg/kg at 28°C. As depicted in Figure 3A, with a temperature increase to 36°C, only 41% (38/93) of the clinical isolates were capable of producing aflatoxin. Within these strains, 55% (23/42) exhibited markedly lower levels of aflatoxin production (*P *< .001), while 29% (12/42) demonstrated enhanced production. The findings provided evidence of temperature-dependent variation in aflatoxin production among *A. flavus* isolates. Other media failed to exhibit the target peaks associated with aflatoxins on the chromatography.

### Aflatoxin biosynthesis-associated gene analysis

**Figure 4.**
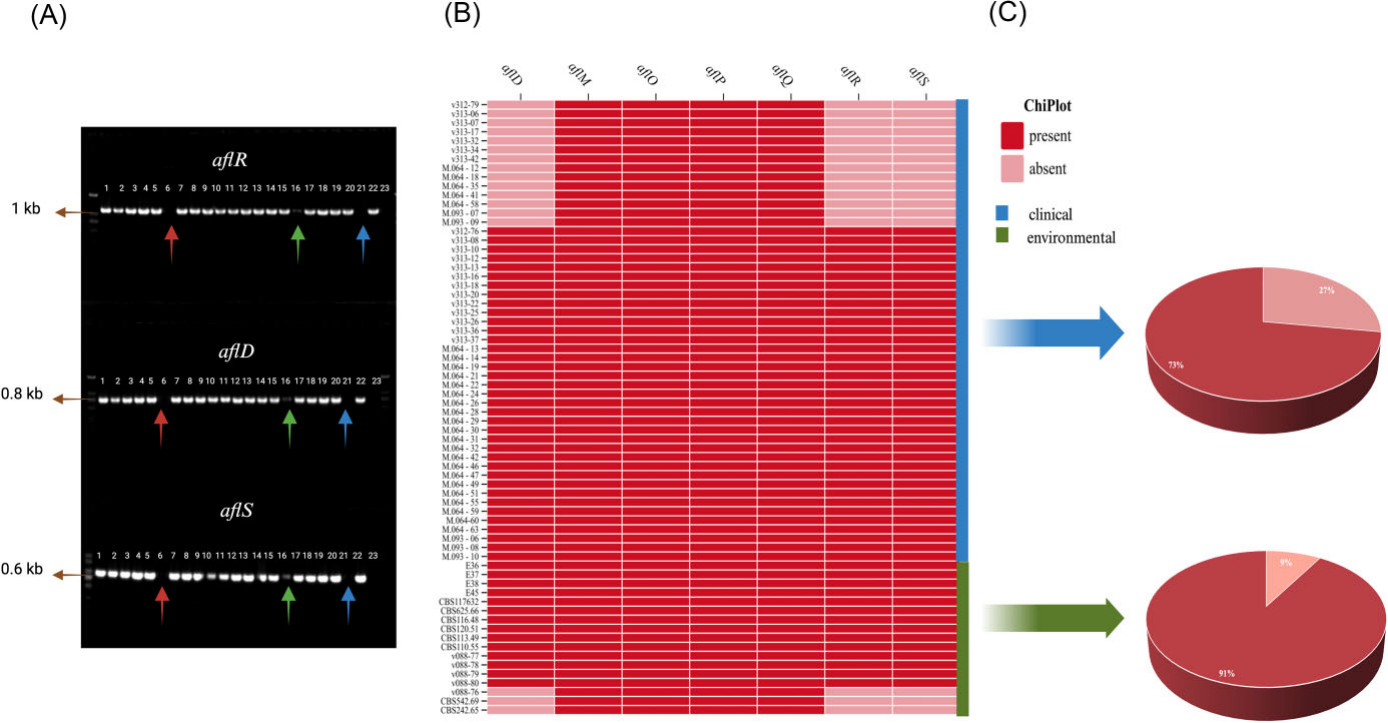
Electrophoresis of PCR results of clinical isolates and the distribution and proportion of aflatoxin biosynthesis-associated genes detected in aflatoxin non-producing isolates. (A) Gel electrophoresis of some isolates (No. 1–22, clinical isolates; No. 23, negative control; No. 6, 16, and 21, *aflD*/*aflR*/*aflS* absence) of *aflD, aflR*, and *aflS*. (B) The distribution of aflatoxin biosynthesis-associated genes detected in aflatoxin non-producing isolates (clinical and environmental isolates). (C) The proportion of *aflD, alfR*, and *aflS* absence and presence.

Seven genes, i.e., *aflD, aflM, aflO, aflP, aflQ, aflR*, and *aflS* were detected. The results showed all aflatoxin-producing isolates including clinical and environmental strains (42/42; 17/17) possessed all seven genes. Figure 4 illustrates the distribution of these genes among the aflatoxin non-producing isolates. Remarkably, among 51 clinical strains and 17 environmental aflatoxin non-producing strains, 37 clinical and 14 environmental isolates were found to harbour all seven genes tested. However, 14 clinical and three environmental strains lacked either *aflD, aflR*, or *aflS* genes (Fig. 4B). Among aflatoxin-non-producing isolates, *aflD, aflR*, and *aflS* were absent in 27% (14/51) of clinical isolates and 9% (3/17) of environmental isolates (Fig. 4C).

To discern the genetic distinctions between aflatoxin-producing and non-producing isolates, targeted sequencing, and alignment of three pivotal aflatoxin biosynthesis-associated genes (*aflD, aflR*, and *aflS*) were conducted. The result is summarized in Table 2. Notably, both aflatoxin-producing and non-producing isolates exhibited mutations within the *aflS* gene in the coding regions. Specifically, two aflatoxin-non-producing isolates displayed a non-synonymous mutation (E193S) in this gene. No mutations were observed in the *aflD* gene of aflatoxin-producing isolates, while a single non-synonymous mutation (A86P) was identified in one aflatoxin-non-producing isolate. Interestingly, mutations within the *aflR* gene were prevalent among the isolates, except for a solitary aflatoxin-non-producing isolate. These mutations in the *aflR* gene were also identified in two aflatoxin-producing isolates.

### Relative gene expression analysis of aflatoxin biosynthesis-associated genes

**Figure 5.**
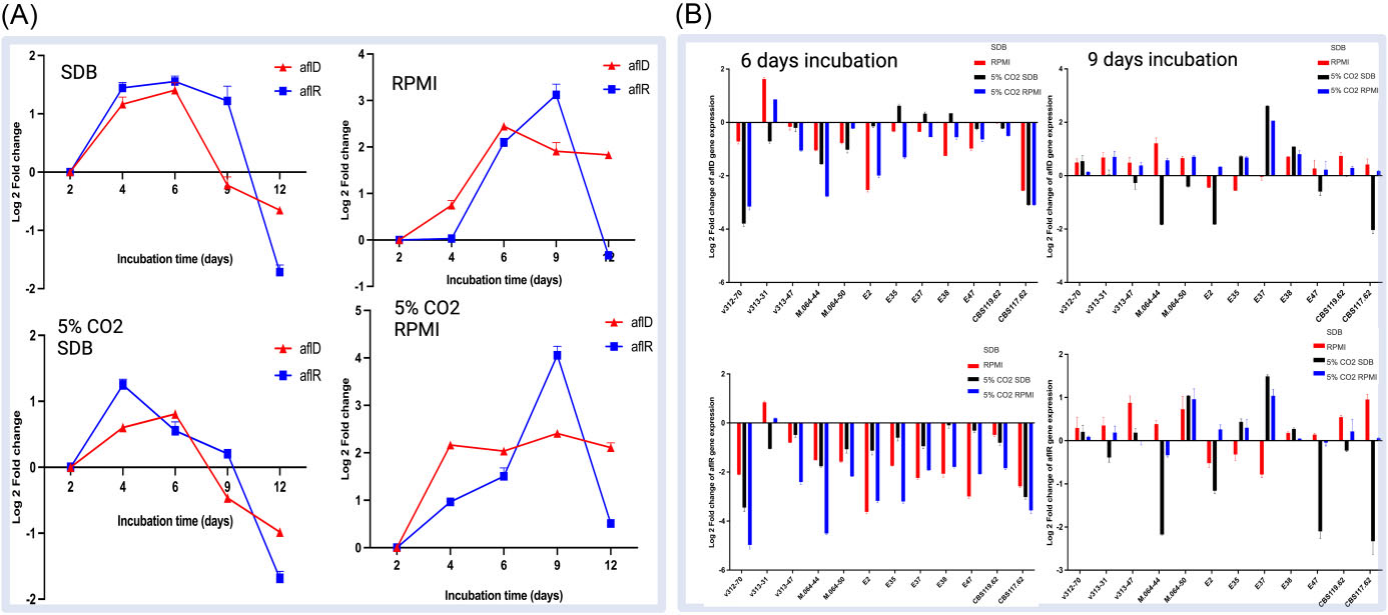
The relative expression of *aflD* and *aflR* of isolate in different media during different incubation time points incubated at 36°C. (A) The relative expression of *aflD* and *aflR* of aflatoxin-producing isolates [the control conditions (calibrator) used for comparisons between treatments 2 days’ incubation]; (B) The relative expression of *aflD* and *aflR* of 10 aflatoxin-producing and two non-producing isolates (E37 and E38) in different media during 6- and 9-days incubation [the control conditions (calibrator) used for comparisons between treatments was the 36°C, SDB, 0.04% CO_2_].

To explore the gene expression trends of *aflD* and *aflR*, four aflatoxin-producing isolates were incubated for 12 days. The results of relative expression analysis of *aflD* and *aflR* are shown in Figure 5A. When 4 aflatoxin-producing isolates were cultured on RPMI medium at 36°C under both 0.04% and 5% CO_2_, a consistent trend emerged for the expression of *aflD* and *aflR* genes over time. Initially, there was an up-regulation, reaching its peak after 9 days of incubation, followed by a sharp decrease. In contrast, for four aflatoxin-producing isolates incubated on SDB medium at 36°C, a different pattern was noted, the expression of both genes reaching its maximum between the 4th and 6th days of incubation and then began to decline. By the 9th day, both genes exhibited downregulation.

To compare *aflD* and *aflR* gene expression of the different types of the medium during 6th and 9th days’ incubation, 12 isolates including each of 10 aflatoxin-producing, and 2 non-producing isolates were assessed (Fig. 5B). The results during the 6-day incubation period revealed that all 12 tested isolates exhibited the downregulated expression of *aflD* and *aflR*, ranging from 0.01 to 3.8 folds when incubated in RPMI, 5% CO_2_ SDB, and 5% CO_2_ RPMI compared to SDB (36°C, 0.04% CO_2_). When 12 isolates were incubated for 9 days in RPMI and 5% CO_2_ RPMI, the expression in 12 isolates showed an upregulation with the range from 0.01 to 2.6 folds compared to SDB (36°C, 0.04% CO_2_).

## Discussion

In our study, characterization of the aflatoxin from 93 isolates obtained from FRS in Sudan and 34 environmental strains was performed. Our results revealed that 45% (42/93) of clinical *Aspergillus flavus* isolates were aflatoxigenic, producing AFB1 and AFB2, similar to the environmental isolates. Furthermore, a significant proportion, i.e., 85% (79/93) of clinical isolates and 91.2% (31/34) of environmental isolates harboured all seven aflatoxin biosynthesis-associated genes.

A comprehensive evaluation of aflatoxin profiles was performed using both phenotypic (TLC and HPLC) and genotypic methods. Using TLC, with its simplicity and cost-effectiveness, served as a preliminary screening tool, we identified aflatoxin presence in 40% of clinical strains. This initial screening prompted further quantitative analysis via HPLC, which unveiled significant variability in aflatoxin concentrations—ranging from 0.01 to 34.17 mg/kg in clinical isolates and from 0.002 to 19.59 mg/kg in environmental samples, suggesting that both clinical and environmental strains possess a significant pathogenic potential.^41,42^ Being an opportunistic pathogen, *A. flavus* infections are primarily acquired through the inhalation of airborne conidia, posing a risk to humans in environments with high aflatoxin levels. The primary intake pathways may include inhalation, ingestion (such as consumption of aflatoxin-contaminated food products) and cutaneous contact (direct contact with contaminated materials).^43^

Aflatoxin production was significantly influenced by our choice of culture medium. Previous research by Liu et al.^44^ demonstrated that *A. flavus* tended to produce higher levels of aflatoxin when cultivated on solid compared to liquid media. Accordingly, SDA was selected as the standard medium for aflatoxin detection in our study, as it provided ample nutrients for fungal growth.^45^ The absence of aflatoxin production in nutrient-poor media (WA) explained that aflatoxin is considered a secondary metabolite, and likely demanded substantial energy investment for biosynthesis.^46^ This process relied on a ready supply of diverse nutrients and precursors, encompassing carbon sources (e.g., sugars), nitrogen sources (e.g., amino acids), as well as vitamins and minerals.^47^ In resource-limited environments, the fungus may prioritize essential metabolic functions necessary for survival and growth, potentially at the expense of secondary metabolite production, such as aflatoxins.^48^ This absence of aflatoxin production is consistent with observations of sporulation degeneration and the presence of thin hyphae in nutrient-poor media.

Our findings also highlighted the relationship between mycotoxin production and sporulation, a phenomenon emphasized by Mostafa et al.^49^ who demonstrated that most of the toxins were produced after the fungus had completed its initial growth phase and began its development stage, characterized by sporulation, and in case of *A. flavus*, sclerotia formation. Existing literature has mentioned that conidia contained a range of secondary metabolites, including melanin and mycotoxins, which played pivotal roles in pathogenesis and development.^50,51^ A series of studies reported aflatoxins using different substrates including natural and synthetic media like potato dextrose agar (PDA), SDA, malt extract agar (MEA), and yeast extract sucrose agar (YES).^45,47^ In contrast, RPMI medium is a common substrate for cell incubation, the first time to be used for analysis of aflatoxin profiles. We encountered a puzzling scenario where aflatoxins were conspicuously absent in the RPMI medium despite the presence of typical conidial heads and dense sporulation during incubation. In contrast to the SDA medium, the RPMI 1640 medium distinguishes itself as the sole option that includes glutathione as a reducing agent and notably higher levels of essential vitamins, including choline, vitamin B12, biotin, and p-aminobenzoic acid (PABA).

Given the limitation of the phenotypic method in detecting aflatoxins and characterizing aflatoxin profiles, the genotypic method emerged as a valuable tool to validate our findings. As emphasized by Dehghan et al., the presence of the *aflR* gene served as a distinctive marker for aflatoxin production in clinical isolates.^52^ This insight provided a critical reference for our current study. Notably, our genotypic result revealed that 85% (79/93) of clinical and 91% of environmental isolates possessed all seven tested genes, indicating the potential risks for aflatoxin production by these isolates. The higher proportion in the environmental isolates possessed all seven tested genes suggested that the host environment, potentially subjected to oxidative stress may stimulated the absence of the major regulators in response to long-term adaption to adverse conditions.^53^ Another study reported that 73% of the isolates contained both *aflR* and *aflD* genes, while 67% possessed the *aflM* gene. This genetic profile was found in both aflatoxigenic and non-aflatoxigenic isolates,^54^ aligning with our findings. However, the result of mutations in aflatoxin biosynthesis-associated genes (*aflD/aflR/aflR*) did not provide clear markers to distinguish aflatoxin-producing and non-producing isolates, as both non-synonymous and synonymous mutations were detected in our study. Existing literature does not provide conclusive insights into this matter. It's essential to clarify that the genotypic method is dedicated to the identification of aflatoxin-related genes, without directly quantifying aflatoxin production. For a precise assessment of specific aflatoxin production, the phenotypic method is employed. Consequently, the integration of both methods is imperative to achieve a comprehensive and research-focused evaluation of the aflatoxin profiles in *A. flavus* isolates.

The gene expression analysis of *aflD* and *aflR* revealed that the highest expression levels occurred between the 4th and 6th day of incubation on the SDB medium and on the 9th day of incubation on the RPMI medium. The variations in substrate led to differences in gene expression levels, possibly explaining why aflatoxins could not be detected by TLC and HPLC in isolates cultured in RPMI for 6 days. It is plausible that the accumulation of aflatoxins was insufficient for detection. This requires further analysis for confirmation. Additionally, a strong correlation between the expression of aflatoxin genes, as analyzed by RT-PCR, and aflatoxin production in 10 aflatoxin-producing isolates was identified. However, two aflatoxin-non-producing strains displayed transcription of all relevant genes but did not produce aflatoxin in the medium, a phenomenon also noted by Degola et al.^55^

In our study, to mimic the host environment and for the first time, 5% CO_2_ was applied to investigate the relative expression of *aflD* and *aflR*. Baazeem et al. explored the effect of climate changes on gene expression and showed that AFB1 production and relative expression of *aflR* was significantly stimulated (*P* < .05) when exposed to 0.1% CO_2_ at 0.98–0.95 aw and 35°C *in vitro*.^25^ We observed that the relative gene expression of *aflR* and *aflD* was down-regulated in 5% CO_2_ in both RPMI and SDB media during 6-day incubation. However, as time progressed, this expression level showed no significant difference beyond 0.04% CO_2_, suggesting that the isolates had adapted to 5% CO_2_ over time. Peromingo et al.^39^ found that gene expression of *aflR* and *aflD* reached its peak when isolates were grown on dry-cured ham during a 6-day incubation period, which was in line with our study when isolates were cultured in an SDB medium. A study conducted by Norlia et al.^56^ has highlighted that environmental factors can potentially facilitate fungal infection. It has been suggested that exposure to host-induced stress may trigger adaptation, leading to gene expression for mycotoxin production and sexual recombination in *A. flavus*. Moreover, Barakat et al.^41^ detected a significant correlation between the degree of severity of keratitis and the quantity of produced AFB1. Hence, in cases of chronic *A. flavus* infection, it is crucial to consider the buildup of aflatoxin. Evaluating the presence or absence of aflatoxins holds significant importance in clinical settings.

Although our study indicated that aflatoxin production depended on the substrate, it is important to note that aflatoxin might be present in the host in cases of aspergillosis. The expression levels of *aflD* and *aflR* varied across different media and time periods, suggesting that accumulation required time. For instance, Klich et al.^57^ reported that *A. flavus* isolates produced aflatoxin in YES but none of them produced toxin on brain heart infusion agar (BHA), a medium that simulates human tissue. However, it's worth noting that the TLC method used may have missed very low levels of toxin. While the authors believed that aflatoxin production is unlikely to play a role in human mycosis, it cannot be ruled out entirely. In 1998, Mori et al.^58^ documented a human case of systemic infection caused by an *A. flavus* isolate that produced aflatoxins both *in vitro* and *in vivo*; extracts from lung lesions and culture filtrates contained aflatoxins B1, B2, and aflatoxin M1. More recently, Mori et al. reported the presence of aflatoxin in the lung tissue of a leukemia patient infected by *A. flavus*.^11^ Although the patient positively responded to leukemia chemotherapy, the primary attribution for their unfortunate demise was *A. flavus* infection and probably the immunosuppressive effects of aflatoxin. Additionally, aflatoxin was detected in the urine of a patient with *A. flavus* pulmonary aspergilloma in Ecuador, although it could not be conclusively established whether the aspergilloma was the definitive source.^59^

The significance of *A. flavus* has been consistently underestimated, despite its prevalence in many regions worldwide and the potential for toxin production by its isolates. It is imperative to draw greater attention to this issue. This necessitates heightened awareness, rigorous assessments of its true impact, and in-depth exploration of the toxin's role in pathogenicity, as well as its possible associations with conditions like nasopharyngeal carcinoma. In light of this, further research and vigilance are warranted to unravel the full extent of its implications and to address the potential health concerns associated with it.

## Conclusions

In conclusion, our study offered comprehensive insights into the characteristics of aflatoxin profiles of *Aspergillus flavus* isolates originating from FRS cases in Sudan. The combined use of phenotypic and genotypic methods was essential for the comprehensive characterization of aflatoxin production profiles. The large number of clinical isolates demonstrated aflatoxigenic capabilities, underscore the potential health risks associated with *A. flavus* infections and the importance of monitoring aflatoxin exposure. A positive correlation between the expression of *aflR* and *aflD*, and aflatoxin production was established, shedding light on the regulation of this mycotoxin. Importantly, dynamic patterns of *aflD* and *aflR* gene expression over time and across different substrates were observed, further enriching our understanding of aflatoxin production regulation. Further research is warranted to elucidate the complex regulation of aflatoxin production and its implications for public health.

## Supplementary Material

myae034_Supplemental_File

## Data Availability

Data will be available on request.

## Fundings

This work was supported by the 111 Project (D20009); China-Ukraine Intergovernmental Exchange Project; National Natural Science Foundation of China (NSFC; no. 32060034); International Science and Technology Cooperation Base of Guizhou Province ([2020]4101); Talent Base Project of Guizhou Province, China [RCJD2018-22]; High-level Innovation Talent Project of Guizhou Province(GCC[2022]036-1); Major Science and Technology Projects of China Tobacco [no. 110202101048(LS-08)]; Foundation of Key Laboratory of Microbiology and Parasitology of Education Department, Guizhou (QJJ [2022] 019); Ministry of Education Project (07150120711); and China Scholarship Council (no. 202108520040).
