# Supplementary material for: Aflatoxin profiles of Aspergillus flavus isolates in Sudanese fungal rhinosinusitis
Source: Med Mycol. 2024 Apr 5;62(4):myae034. doi: 10.1093/mmy/myae034 (PMC11040519; doi:10.1093/mmy/myae034)
Supplement: myae034_Supplemental_File [file myae034_supplemental_file.docx]

S1 The information of environmental and some clinical isolates

| Number | Strains | species | Isolation places and substrates | GenBank accession number | | |
| --- | --- | --- | --- | --- | --- | --- |
|  |  |  |  | *BT2* | *CaM* | ITS |
| 1 | M.093 - 12 | *Aspergillus flavus* | Hebei, corn | PP493351 | PP496538 | - |
| 2 | M.093 - 13 | *Aspergillus flavus* | Hebei, corn | PP493352 | PP496539 | - |
| 3 | M.093 - 14 | *Aspergillus flavus* | Shanghai, corn | PP493353 | PP496540 | - |
| 4 | M.093 - 15 | *Aspergillus flavus* | Shanghai, corn | PP493354 | PP496541 | - |
| 5 | M.093 - 16 | *Aspergillus flavus* | Guangzhou, corn | PP493355 | PP496542 | - |
| 6 | M.093 - 17 | *Aspergillus flavus* | Guangzhou, corn | PP493356 | PP496542 | - |
| 7 | M.093 - 18 | *Aspergillus flavus* | Hangzhou,corn | PP493357 | PP496544 | - |
| 8 | M.093 - 19 | *Aspergillus flavus* | Hangzhou,corn | PP493358 | PP496545 | - |
| 9 | M.093 - 20 | *Aspergillus flavus* | Henan, corn | PP493359 | PP496546 | - |
| 10 | M.093 - 21 | *Aspergillus flavus* | Changzhi, corn | PP493360 | PP496547 | - |
| 11 | M.093 - 22 | *Aspergillus flavus* | Hefei, peanut | PP493361 | PP496538 | - |
| 12 | M.093 - 23 | *Aspergillus flavus* | Hefei, peanut | PP493362 | PP496548 | - |
| 13 | M.093 - 26 | *Aspergillus flavus* | Guizhou, peanut | PP493363 | PP496549 | - |
| 14 | M.093 - 27 | *Aspergillus flavus* | Guizhou, peanut | PP493364 | PP496550 | - |
| 15 | M.093 - 28 | *Aspergillus flavus* | Kunming, peanut | PP493365 | PP496551 | - |
| 16 | M.093 - 29 | *Aspergillus flavus* | Kunming, peanut | PP493366 | PP496552 | - |
| 17 | CBS 119.62 | *Aspergillus flavus var. flavus* | Brazil, Arachis hypogaea | MH878538.1  (LSU) | | |
| 18 | CBS 816.96 | *Aspergillus flavus var. flavus* | Sweden | KJ175478.1 | KJ175533.1 | KJ175413.1 |
| 19 | CBS 117.62 | *Aspergillus flavus var. flavus* | Uganda, Arachis hypogaea | MH869691  (LSU) | | |
| 20 | CBS 118.62 | *Aspergillus flavus* | Brazil, Arachis hypogaea | - | - | - |
| 21 | CBS 501.65 | *Aspergillus subolivaceus* | England, cotton Lintafelt | EF203144.1 | EF202064.1 | - |
| 22 | CBS 117632 | *Aspergillus flavus* | Argentina, Arachis hypogaea, seed | EF203137.1 | EF202058.1 | - |
| 23 | CBS 625.66 | *Aspergillus flavus var. flavus* | Papua New Guinea, shoe | - | - | - |
| 24 | CBS 116.48 | *Aspergillus flavus var. flavus* | Unknown | EF203141.1 | EF202054.1 | - |
| 25 | CBS 120.51 | *Aspergillus flavus* | England, culture contaminant | EF203133.1 | EF202070.1 | FJ491466.1 |
| 26 | CBS 113.49 | *Aspergillus flavus var. flavus* | Java, air | - | - | - |
| 27 | CBS 110.55 | *Aspergillus flavus* | Brazil, air contaminant | EF203135.1 | - | MH857396.1 |
| 28 | CBS 542.69 | *Aspergillus flavus* | Japan, stratigraphic core sample | EF203136.1 | EF202069.1 | MH859373.1 |
| 29 | CBS 242.65 | *Aspergillus flavus var. columnaris* | South Africa,commeal | - | - | MH858555.1 |
| 30 | v088-76 | *Aspergillus flavus* | Netherland, environment | PP493367 | PP497029 | - |
| 31 | v088-77 | *Aspergillus flavus* | Netherland, environment | PP493368 | PP497030 | - |
| 32 | v088-78 | *Aspergillus flavus* | Netherland, environment | PP493369 | PP497031 | - |
| 33 | v088-79 | *Aspergillus flavus* | Netherland, environment | PP493370 | PP497032 | - |
| 34 | v088-80 | *Aspergillus flavus* | Netherland, environment | PP4933671 | PP497033 | - |
| 35 | M.093-6 | *Aspergillus flavus* | Sudan, sinus | PP493346 | PP497008 | - |
| 36 | M.093-7 | *Aspergillus flavus* | Sudan, sinus | PP493347 | PP497009 |  |
| 37 | M.093-8 | *Aspergillus flavus* | Sudan, sinus | PP493348 | PP497010 | - |
| 38 | M.093-9 | *Aspergillus flavus* | Sudan, sinus | PP493349 | PP497011 | - |
| 39 | M.093-10 | *Aspergillus flavus* | Sudan, sinus | PP493350 | PP497012 | - |
| 40 | M.093-24 | *Penicillium citrinum* | Hefei, peanut | - | - | PP496537 |
| 41 | M.093-25 | *Trichoderma asperellum* | Guizhou, peanut | - | - | PP496554 |
| 42 | M.064-61 | *Aspergillus terreus* | Sudan, sinus | OR451370 | OR451276 | - |

“-” represents unavailable.

S2 The linear regression equations, linear ranges, correlation coefficients, LOD and LQD for aflatoxins B1, B2, G1 and G2

| Aflatoxins | Linear range (ng/mL) | Linear regression equation | R² | LOD (ng/g) | LQD（ng/g） |
| --- | --- | --- | --- | --- | --- |
| AFB2 | 0.15-3 | y = 9317.3x - 1221 | 0.9991 | 0.07 | 0.22 |
| AFB1 | 0.5-10 | y = 1683.8x - 737.06 | 0.999 | 0.22 | 0.72 |
| AFG2 | 0.15-3 | y = 6504.8x - 722.66 | 0.9995 | 0.06 | 0.18 |
| AFG1 | 0.5-10 | y = 926.23x - 434.36 | 0.9992 | 0.23 | 0.77 |

Abbreviation: R^2^-Correlation coefficients; LOD- detection limit; LQD- quantification limit

S3 The GenBank accession number of *afl* gene clusters

| **Origin** | **Number** | **species** | ***aflD*** | ***aflS*** |
| --- | --- | --- | --- | --- |
| 1-4 | v312-70 | *Aspergillus flavus* | PP493372 | PP496821 |
| 1-7 | v312-73 | *Aspergillus flavus* | PP493373 | PP496822 |
| 1-8 | v312-74 | *Aspergillus flavus* | PP493374 | PP496823 |
| 1-62 | v313-47 | *Aspergillus flavus* | PP493375 | PP496824 |
| 2-34 | M.064-44 | *Aspergillus flavus* | PP493376 | PP496825 |
| 1-10 | v312-76 | *Aspergillus flavus* | PP493377 | PP496826 |
| 1-25 | v313-10 | *Aspergillus flavus* | PP493378 | PP496827 |
| 1-27 | v313-12 | *Aspergillus flavus* | PP493379 | PP496828 |
| 2-44 | M.064-55 | *Aspergillus flavus* | PP493380 | PP496829 |

**S4 Aflatoxins detected by TLC and HPLC**

| **Clinical isolates** | **Radboudumc ID** | **Number of isolates** | **TLC detetion** | **AFB（mg/kg）** | | | | | |
| --- | --- | --- | --- | --- | --- | --- | --- | --- | --- |
|  |  |  |  | **28 ℃** | | | **36 ℃** | | |
|  |  |  |  | **Total AFB** | **B1** | **B2** | **Total AFB** | **B1** | **B2** |
| 1 | v312-70 | 1-4 | + | 12.53±0.03 | 10.54±0.04 | 1.99±0.05 | 0.51±0.01 | 0.5±0.01 | 0.01±0 |
| 2 | v312-73 | 1-7 | + | 4.2±0.02 | 3.11±0.01 | 1.09±0.03 | 0.1±0 | 0.1±0 | - |
| 3 | v312-74 | 1-8 | + | 9.61±0.03 | 7.91±0.03 | 1.7±0.04 | 0.04±0 | 0.04±0 | - |
| 4 | v312-77 | 1-11 | + | 1.69±0 | 1.26±0.01 | 0.43±0.01 | 0.12±0 | 0.11±0 | 0.01±0 |
| 5 | v313-09 | 1-24 | + | 2.05±0.02 | 1.57±0.01 | 0.48±0.01 | 0.01±0 | 0.01±0 | - |
| 6 | v313-11 | 1-26 | + | 2.36±0.01 | 1.85±0 | 0.51±0.01 | 0.11±0 | 0.11±0 | - |
| 7 | v313-15 | 1-30 | + | 2.53±0.01 | 1.79±0 | 0.74±0.02 | 0.17±0 | 0.16±0 | 0.01±0 |
| 8 | v313-28 | 1-43 | + | 5.81±0.04 | 4.1±0.01 | 1.71±0.03 | 1.2±0.02 | 1.2±0.02 | - |
| 9 | v313-29 | 1-44 | + | 3.56±0.02 | 2.65±0 | 0.9±0.02 | 0.56±0 | 0.54±0 | 0.02±0 |
| 10 | v313-30 | 1-45 | + | 7.99±0.02 | 6.82±0.01 | 1.17±0.02 | 4.71±0.06 | 4.37±0.06 | 0.34±0 |
| 11 | v313-31 | 1-46 | + | 14.32±0.11 | 9.06±0.09 | 5.27±0.13 | 0.28±0.01 | 0.28±0.01 | 0.01±0 |
| 12 | v313-41 | 1-56 | + | 1.45±0 | 1.1±0.01 | 0.35±0.01 | 0.06±0 | 0.06±0 | - |
| 13 | v313-44 | 1-59 | + | 8.24±0.04 | 6.34±0 | 1.89±0.04 | 8.36±0.02 | 8±0.02 | 0.36±0 |
| 14 | v313-47 | 1-62 | + | 8.3±0.04 | 6.68±0 | 1.62±0.04 | 20.75±0.13 | 20.12±0.12 | 0.64±0 |
| 15 | M.064 - 17 | 2-7 | + | 0.85±0.02 | - | 0.85±0.02 | 1.66±0.06 | 1.66±0.06 | - |
| 16 | M.064 - 33 | 2-23 | + | 2.54±0.03 | 1.27±0 | 1.27±0.03 | 2.72±0.02 | 2.72±0.02 | - |
| 17 | M.064 - 34 | 2-24 | + | 1.76±0.01 | 1.19±0 | 0.58±0.01 | - | - | - |
| 18 | M.064 - 37 | 2-27 | + | 10.26±0.01 | 9.99±0 | 0.26±0 | 3.69±0.02 | 3.69±0.02 | - |
| 19 | M.064 - 44 | 2-34 | + | 12.77±0 | 12.42±0.01 | 0.35±0.01 | 17.9±0.06 | 17.42±0.06 | 0.47±0 |
| 20 | M.064 - 50 | 2-39 | + | 10.12±0.02 | 9.66±0.01 | 0.46±0.01 | 10.63±0.02 | 10.23±0.02 | 0.4±0.01 |
| 21 | M.064 - 52 | 2-41 | + | 34.17±0 | 33.83±0.01 | 0.35±0.01 | 8.54±0.02 | 8.22±0.03 | 0.32±0.01 |
| 22 | M.064 - 53 | 2-42 | + | 9.24±0 | 8.88±0.01 | 0.36±0.01 | - | - | - |
| 23 | M.064 - 54 | 2-43 | + | 10.69±0.01 | 10.11±0 | 0.58±0.01 | 0.22±0 | 0.21±0 | 0.01±0 |
| 24 | M.064 - 57 | 2-46 | + | 8.79±0.03 | 8.46±0.04 | 0.33±0.01 | 1.78±0.03 | 1.78±0.03 | - |
| 25 | M.064 - 43 | 2-33 | - | 3±0.06 | 0.71±0 | 2.29±0.06 | - | - | - |
| 26 | v313-35 | 1-50 | + | 1.09±0 | 0.76±0.01 | 0.32±0.01 | 0.15±0 | 0.15±0 | 0.001±0 |
| 27 | v313-38 | 1-53 | + | 0.27±0 | 0.16±0 | 0.11±0 | 1.58±0 | 1.51±0 | 0.07±0 |
| 28 | M.064 - 20 | 2-10 | + | 0.17±0 | 0.13±0 | 0.04±0 | 0.09±0 | 0.09±0 | - |
| 29 | M.064 - 36 | 2-26 | + | 0.87±0 | 0.82±0 | 0.04±0 | 0.11±0 | 0.11±0 | - |
| 30 | M.064 - 39 | 2-29 | + | 0.18±0 | 0.18±0 | - | 0.17±0 | 0.17±0 | - |
| 31 | M.064 - 40 | 2-30 | + | 0.32±0 | 0.32±0 | - | 0.32±0 | 0.32±0 | - |
| 32 | M.064 - 45 | 2-35 | + | 0.39±0 | 0.39±0 | - | 0.38±0.01 | 0.38±0.01 | - |
| 33 | v313-03 | 1-18 | - | 0.04±0 | 0.02±0 | 0.02±0 | 1.62±0.03 | 1.6±0.03 | 0.02±0 |
| 34 | v313-14 | 1-29 | + | 0.03±0 | - | 0.03±0 | 0.31±0 | 0.3±0 | 0.01±0 |
| 35 | v313-33 | 1-48 | - | 0.06±0 | 0.04±0 | 0.02±0 | 4.94±0.04 | 4.59±0.04 | 0.35±0 |
| 36 | v313-39 | 1-54 | - | 0.01±0.02 | 0±0 | 0.01±0.02 | 0.03±0 | 0.03±0 | - |
| 37 | v313-40 | 1-55 | - | 0.12±0.06 | 0.03±0 | 0.08±0.06 | 0.05±0 | 0.03±0 | 0.01±0 |
| 38 | M.064 - 11 | 2-1 | + | 0.12±0 | 0.1±0 | 0.02±0 | 1.14±0.07 | 1.14±0.07 | - |
| 39 | M.064 - 23 | 2-13 | - | 0.01±0 | - | 0.01±0 | - | - | - |
| 40 | M.064 - 25 | 2-15 | + | 0.02±0 | - | 0.02±0 | 11.06±0.04 | 10.67±0.03 | 0.39±0.01 |
| 41 | M.064 - 27 | 2-17 | + | 0.04±0 | - | 0.04±0 | 1.35±0.02 | 1.35±0.02 | - |
| 42 | M.064 - 38 | 2-28 | - | 0.03±0 | 0.03±0 | - | 0.03±0 | 0.03±0 | - |
| 1 | v312-79 | 1-13 | - | - | - | - | - | - | - |
| 2 | v313-06 | 1-21 | - | - | - | - | - | - | - |
| 3 | v313-07 | 1-22 | - | - | - | - | - | - | - |
| 4 | v313-17 | 1-32 | - | - | - | - | - | - | - |
| 5 | v313-32 | 1-47 | - | - | - | - | - | - | - |
| 6 | v313-34 | 1-49 | - | - | - | - | - | - | - |
| 7 | v313-42 | 1-57 | - | - | - | - | - | - | - |
| 8 | M.064 - 12 | 2-2 | - | - | - | - | - | - | - |
| 9 | M.064 - 18 | 2-8 | - | - | - | - | - | - | - |
| 10 | M.064 - 35 | 2-25 | - | - | - | - | - | - | - |
| 11 | M.064 - 41 | 2-31 | - | - | - | - | - | - | - |
| 12 | M.064 - 58 | 2-47 | - | - | - | - | - | - | - |
| 13 | M.093 - 07 | P2 | - | - | - | - | - | - | - |
| 14 | M.093 - 09 | P4 | - | - | - | - | - | - | - |
| 1 | v312-76 | 1-10 | - | - | - | - | - | - | - |
| 2 | v313-08 | 1-23 | - | - | - | - | - | - | - |
| 3 | v313-10 | 1-25 | - | - | - | - | - | - | - |
| 4 | v313-12 | 1-27 | - | - | - | - | - | - | - |
| 5 | v313-13 | 1-28 | - | - | - | - | - | - | - |
| 6 | v313-16 | 1-31 | - | - | - | - | - | - | - |
| 7 | v313-18 | 1-33 | - | - | - | - | - | - | - |
| 8 | v313-20 | 1-35 | - | - | - | - | - | - | - |
| 9 | v313-22 | 1-37 | - | - | - | - | - | - | - |
| 10 | v313-25 | 1-40 | - | - | - | - | - | - | - |
| 11 | v313-26 | 1-41 | - | - | - | - | - | - | - |
| 12 | v313-36 | 1-51 | - | - | - | - | - | - | - |
| 13 | v313-37 | 1-52 | - | - | - | - | - | - | - |
| 14 | M.064 - 13 | 2-3 | - | - | - | - | - | - | - |
| 15 | M.064 - 14 | 2-4 | - | - | - | - | - | - | - |
| 16 | M.064 - 19 | 2-9 | - | - | - | - | - | - | - |
| 17 | M.064 - 21 | 2-11 | - | - | - | - | - | - | - |
| 18 | M.064 - 22 | 2-12 | - | - | - | - | - | - | - |
| 19 | M.064 - 24 | 2-14 | - | - | - | - | - | - | - |
| 20 | M.064 - 26 | 2-16 | - | - | - | - | - | - | - |
| 21 | M.064 - 28 | 2-18 | - | - | - | - | - | - | - |
| 22 | M.064 - 29 | 2-19 | - | - | - | - | - | - | - |
| 23 | M.064 - 30 | 2-20 | - | - | - | - | - | - | - |
| 24 | M.064 - 31 | 2-21 | - | - | - | - | - | - | - |
| 25 | M.064 - 32 | 2-22 | - | - | - | - | - | - | - |
| 26 | M.064 - 42 | 2-32 | - | - | - | - | - | - | - |
| 27 | M.064 - 46 | 2-36 | - | - | - | - | - | - | - |
| 28 | M.064 - 47 | 2-37 | - | - | - | - | - | - | - |
| 29 | M.064 - 49 | 2-38 | - | - | - | - | - | - | - |
| 30 | M.064 - 51 | 2-40 | - | - | - | - | - | - | - |
| 31 | M.064 - 55 | 2-44 | - | - | - | - | - | - | - |
| 32 | M.064 - 59 | 2-48 | - | - | - | - | - | - | - |
| 33 | M.064-60 | 2-49 | - | - | - | - | - | - | - |
| 34 | M.064 - 63 | 2-52 | - | - | - | - | - | - | - |
| 35 | M.093 - 06 | P1 | - | - | - | - | - | - | - |
| 36 | M.093 - 08 | P3 | - | - | - | - | - | - | - |
| 37 | M.093 - 10 | P5 | - | - | - | - | - | - | - |
| Environmental isolates | **Radboudumc ID** | **Number of isolates** | **TLC detetion** | **AFB（mg/kg）** | | | | | |
|  |  |  |  | **28 ℃** | | | **36 ℃** | | |
|  |  |  |  | **Total AFB** | **B1** | **B2** | **Total AFB** | **B1** | **B2** |
| 1 | M.093 - 12 | E1 | + | 0.53±0.01 | 0.51±0.01 | 0.02±0 |  |  |  |
| 2 | M.093 - 13 | E2 | + | 19.59±0.22 | 19.37±0.22 | 0.23±0 |  |  |  |
| 3 | M.093 - 14 | E5 | + | 1.4±0.01 | 1.38±0 | 0.02±0 |  |  |  |
| 4 | M.093 - 15 | E6 | + | 2.04±0.05 | 2.02±0.05 | 0.02±0 |  |  |  |
| 5 | M.093 - 16 | E13 | + | 0.6±0.71 | 0.6±0.71 | 0.01±0 |  |  |  |
| 6 | M.093 - 17 | E31 | + | 2.98±0.01 | 2.98±0.01 | - |  |  |  |
| 7 | M.093 - 18 | E33 | + | 1.87±0.04 | 1.87±0.04 | - |  |  |  |
| 8 | M.093 - 19 | E34 | + | 2.36±0 | 2.23±0 | 0.13±0 |  |  |  |
| 9 | M.093 - 20 | E35 | + | 7.62±0.01 | 7.28±0.01 | 0.35±0 |  |  |  |
| 10 | M.093 - 27 | E47 | + | 4.45±0 | 4.33±0.01 | 0.12±0 |  |  |  |
| 11 | M.093 - 28 | E48 | + | 0.74±0 | 0.72±0 | 0.02±0 |  |  |  |
| 12 | M.093 - 29 | E49 | + | 0.65±0 | 0.63±0 | 0.02±0 |  |  |  |
| 13 | M.093 - 30 | CBS119.62 | + | 0.05±0 | 0.02±0 | 0.02±0 |  |  |  |
| 14 | M.093 - 31 | CBS816.96 | + | 0.04±0 | 0.04±0 | 0.003±0 |  |  |  |
| 15 | M.093 - 35 | CBS117.62 | + | 0.37±0 | 0.36±0 | 0.003±0 |  |  |  |
| 16 | M.093 - 33 | CBS118.62 | + | 0.45±0 | 0.45±0 | 0.003±0 |  |  |  |
| 17 | M.093 - 34 | CBS501.65 | + | 0.002±0 | 0.002±0 | - |  |  |  |
| 1 | M.093 - 21 | E36 | - | - | - | - |  |  |  |
| 2 | M.093 - 22 | E37 | - | - | - | - |  |  |  |
| 3 | M.093 - 23 | E38 | - | - | - | - |  |  |  |
| 4 | M.093 - 26 | E45 | - | - | - | - |  |  |  |
| 5 | M.093 - 32 | CBS117632 | - | - | - | - |  |  |  |
| 6 | M.093 - 36 | CBS625.66 | - | - | - | - |  |  |  |
| 7 | M.093 - 37 | CBS116.48 | - | - | - | - |  |  |  |
| 8 | M.093 - 38 | CBS120.51 | - | - | - | - |  |  |  |
| 9 | M.093 - 39 | CBS113.49 | - | - | - | - |  |  |  |
| 10 | M.093 - 40 | CBS110.55 | - | - | - | - |  |  |  |
| 11 | v088-77 | v088-77 | - | - | - | - |  |  |  |
| 12 | v088-78 | v088-78 | - | - | - | - |  |  |  |
| 13 | v088-79 | v088-79 | - | - | - | - |  |  |  |
| 14 | v088-80 | v088-80 | - | - | - | - |  |  |  |
| 15 | v088-76 | v088-76 | - | - | - | - |  |  |  |
| 16 | M.093 - 41 | CBS542.69 | - | - | - | - |  |  |  |
| 17 | M.093 - 42 | CBS242.65 | - | - | - | - |  |  |  |

Abbreviation: TLC thin layer chromatography; HPLC high performance liquid chromatography; AFB aflatoxin B; + aflatoxin positive; - aflatoxin negative; blank no detection.
